# Association between a Genetic Variant of Type-1 Cannabinoid Receptor and Inflammatory Neurodegeneration in Multiple Sclerosis

**DOI:** 10.1371/journal.pone.0082848

**Published:** 2013-12-31

**Authors:** Silvia Rossi, Marco Bozzali, Monica Bari, Francesco Mori, Valeria Studer, Caterina Motta, Fabio Buttari, Mara Cercignani, Paolo Gravina, Nicolina Mastrangelo, Maura Castelli, Raffaele Mancino, Carlo Nucci, Fabrizio Sottile, Sergio Bernardini, Mauro Maccarrone, Diego Centonze

**Affiliations:** 1 Clinica Neurologica, Dipartimento di Medicina dei Sistemi, Università Tor Vergata, Rome, Italy; 2 Centro Europeo per la Ricerca sul Cervello (CERC)/Fondazione Santa Lucia, Rome, Italy; 3 Dipartimento di Medicina Sperimentale e Chirurgia, Università Tor Vergata, Rome, Italy; 4 Clinical Imaging Sciences Centre, Brighton and Sussex Medical School, University of Sussex, Brighton, East Sussex, United Kingdom; 5 Dipartimento Medicina di Laboratorio, Policlinico Tor Vergata, Rome, Italy; 6 Clinica Oculistica, Dipartimento di Biopatologia, Università Tor Vergata, Rome, Italy; 7 IRCCS Centro Neurolesi Bonino Pulejo, Messina, Italy; 8 Dipartimento di Medicina Interna, Università Tor Vergata, Rome, Italy; 9 Center of Integrated Research, School of Medicine, Campus Bio-Medico University of Rome, Rome, Italy; University Hospital La Paz, Spain

## Abstract

Genetic ablation of type-1 cannabinoid receptors (CB_1_Rs) exacerbates the neurodegenerative damage of experimental autoimmune encephalomyelitis, the rodent model of multiple sclerosis (MS). To address the role on CB_1_Rs in the pathophysiology of human MS, we first investigated the impact of AAT trinucleotide short tandem repeat polymorphism of CNR1 gene on CB_1_R cell expression, and secondly on the inflammatory neurodegeneration process responsible for irreversible disability in MS patients. We found that MS patients with long AAT repeats within the CNR1 gene (≥12 in both alleles) had more pronounced neuronal degeneration in response to inflammatory white matter damage both in the optic nerve and in the cortex. Optical Coherence Tomography (OCT), in fact, showed more severe alterations of the retinal nerve fiber layer (RNFL) thickness and of the macular volume (MV) after an episode of optic neuritis in MS patients carrying the long AAT genotype of CNR1. MS patients with long AAT repeats also had magnetic resonance imaging (MRI) evidence of increased gray matter damage in response to inflammatory lesions of the white matter, especially in areas with a major role in cognition. In parallel, visual abilities evaluated at the low contrast acuity test, and cognitive performances were negatively influenced by the long AAT CNR1 genotype in our sample of MS patients. Our results demonstrate the biological relevance of the (AAT)_n_ CNR1 repeats in the inflammatory neurodegenerative damage of MS.

## Introduction

Type-1 cannabinoid receptors (CB_1_Rs) are among the most abundant G protein-coupled receptors in the mammalian brain [1]–[3], where they play a pivotal role in the control of synaptic transmission [4], and in the maintenance of neuronal integrity [5], [6]. Not surprisingly, cannabinoid treatment has been proposed to contrast the neurodegenerative damage in several neuroinflammatory diseases [7]–[9]. Genetic ablation of CB_1_Rs exacerbates the neurodegenerative damage associated with experimental autoimmune encephalomyelitis (EAE), a reliable mouse model of multiple sclerosis (MS), by altering synaptic sensitivity to pro-inflammatory cytokines released by infiltrating immune cells and by activated microglia [10]–[12]. Inflammation leads to neuronal damage also in the human brain, and indeed higher frequency and severity of inflammatory episodes have been associated with accelerated neurodegeneration and disability accumulation in MS [13]–[15], but large inter-individual differences among patients exist. Based on this clinical evidence, we postulated therefore that genetic differences in CB_1_R expression and function might contribute to differential inflammatory neurodegenerative damage in MS patients, as it occurs in EAE mice.

The gene encoding CB_1_R (CNR1) is located on chromosome 6, and shows a microsatellite polymorphism which is an AAT trinucleotide short tandem repeat (AAT)_n_, downstream of the translation site [16]. There is evidence indicating that microsatellites can affect transcription efficacy in some genes [17], and if true also for CNR1, this notion offers the unprecedented opportunity to address our hypothesis. Of note, although in a previous study [18] some clinical measures of disease severity were unaffected by this microsatellite polymorphism, MS patients with primary progressive disease course were found to have more commonly long AAT repeats, in line with the idea that neurodegenerative damage can be influenced in MS by CB_1_Rs [18].

Thus, here we first investigated the impact of (AAT)_n_ CNR1 repeats on CB_1_R expression, and then on the inflammatory neurodegeneration processes responsible for irreversible disability in MS patients. Our results provide initial evidence that long (≥12 in both alleles) AAT repeats within the CNR1 gene reduce CB_1_R expression in MS patients, and exacerbate the impact of inflammation on neuronal integrity and function in the optic nerve and in the brain of MS patients.

## Materials and Methods

This study complied with the principles of the Declaration of Helsinki, and was approved by the Ethical Committee of the Policlinico Università Tor Vergata in Rome. All subjects gave their written informed consent.

### MS subjects

A total of 114 central-southern Italian subjects were included in this study. All had a diagnosis of relapsing-remitting MS [19]. MS disease onset was defined as the first episode of focal neurological dysfunction indicative of MS. Relapses were defined as the development of new or recurrent neurological symptoms not associated with fever or infection lasting for at least 24 h. Disease duration was estimated as the number of years from onset to the last assessment of disability.

At the time of confirmed diagnosis, all MS patients had started disease-modifying therapy (glatiramer acetate 20 mg s.c. daily, interferon beta 1a 44 mcg s.c. three times weekly, interferon beta 1a 30 mcg i.m., or interferon beta 1b 250 mcg s.c. every other day). Mitoxantrone (12 mg/m^2^ i.v. every 3 months with a life-time maximum of 140 mg/m^2^) and natalizumab (300 mg i.v. every four weeks) were considered as second-line treatments.

### Determination of AAT repeats in the CNR1 gene

Peripheral blood samples of MS patients were collected in BD Vacutainer tubes containing EDTA (Beckton Dickinson, Franklin Lakes, NJ). Genomic DNA was purified from 200 µl of human whole blood using MagNA Pure LC DNA Isolation Kit (Roche Diagnostics GmbH, Mannheim, Germany) in an automated extractor MagNA Pure LC (Roche Diagnostics) according to the manufacturer's instructions. The CNR1 region containing the AAT repeats was amplified by polymerase chain reaction (PCR) from 150 ng of genomic DNA. PCR reaction was performed in a final volume of 25 µl containing polymerase buffer, 1 mM MgCl_2_, 0.2 mM of each dNTP, 0.5 pmoles of each primer (sense: 5′-CACCCCTGGGCTGTAAAATA-3′; antisense: 5′-GTTGCAGTGAGCCAAGATCA-3′) and 1.5 U Taq DNA polymerase (Invitrogen, Madison, USA). Amplification reaction consisted in an initial denaturation step at 94°C for 5 minutes, followed by 35 cycles of denaturation at 95°C for 45″, annealing at 58°C for 1.5 minutes and elongation at 72°C for 1′, and a final elongation step at 72°C for 7′. Sequencing analysis were performed from 10 ng of PCR products, purified with Agencourt AMPure PCR Purification kit (Agencourt Bioscience Corporation, Beverly, MA) in accordance with manufacturer's instructions, using 0.5 pmoles of the sequencing primer (5′-ACCTCCACCCACAAATCAAA-3′) and the ABI PRISM BigDye Terminator v3.1 Ready Reaction Cycle Sequencing Kit (Applied Biosystems, Foster City, CA). Sequencing reactions consisted in an initial denaturation step at 96°C for 1 minute, followed by 40 cycles at 96°C for 10 seconds, 50°C for 5 seconds, and 60°C for 4 minutes. Sequencing products were purified using CleanSEQ dye terminal removal kit (Agencourt Bioscience Corporation) in accordance with manufacturer's instructions and run on the Applied Biosystems 3730 DNA Analyzer Instrument (Applied Biosystems). AAT repeats were counted on the resulting electropherograms.

### Determination of CB_1_R protein expression

In a recent study we have demonstrated that rabbit anti-CB_1_R antibodies (cat. 101500; Cayman Chemical Co., Ann Arbor, MI, USA) recognize a specific band of the expected molecular mass of CB_1_R in human peripheral lymphocytes, subjected to 10% SDS-PAGE and electroblotting [20]. Specificity of the anti-CB_1_R antibodies was ascertained by preincubating 1 µg of them with 10 µg of the specific blocking peptide (Cayman Chemical Co.), that was able to fully erase the immunoreactive band [20]. Here, the same anti-CB_1_R antibodies were used in quantitative enzyme-linked immunosorbent assays (ELISA), performed on whole cell lysates (20 µg/well). In addition, in order to draw dose-response curves and ascertain the linearity range of the ELISA test, different amounts of human CB_1_R-transfected Chinese hamster ovarian cells (CHO-CB_1_, from Millipore, Bedford. MA, USA) were analyzed as reported [21]. Briefly, CHO-CB_1_ extracts (in the protein range 0–40 µg/well) were incubated with primary anti-CB_1_R antibodies (1∶500); in negative controls, the same antibodies were pre-incubated with the specific CB_1_ blocking peptide (1∶10 ratio) for 3 h at room temperature. After incubation with alkaline phosphatase-conjugated secondary antibody (1∶2000 dilution; Bio-Rad, Hercules, CA, USA), color development of the alkaline phosphatase reaction was measured at 405 nm, using p-nitrophenyl phosphate as substrate [21]. A_405_ values of lymphocyte extracts were always within the linearity range of the calibration curves drawn with CHO-CB_1_R cell homogenates, and were used to estimate CB_1_R content in human lymphocytes.

### Ophthalmologic assessment

Medical history with respect to visual symptoms was taken from all MS subjects. Self-report and physician report were confirmed by record review.

A sample of MS patients with no history of ophthalmological disease (n = 70) underwent measurement of retinal nerve fiber layer (RNFL) thickness, Macular Volume (MV) for both eyes using Stratus Optical Coherence Tomography ([OCT™] software version 4.0.2, Carl Zeiss Meditec, Inc.). Briefly, for MV, retinal thickness was measured automatically as the distance between the vitreoretinal interface and the anterior boundary of the retinal pigment epithelium. Stratus OCT images were generated using the fast map scan protocol consisting of six radial scans spaced 30° apart, with each scan measuring 6 mm in length. Each image had a resolution of 10 µm axially and 20 µm transversally. All Stratus OCT images had a signal strength of 6 µm. RNFL thickness measurements were read from the automated measurements generated by the machine using the Fast RNFL analysis. For the study scanning was performed after pharmacological dilation. Average RNFL thickness for 360° around the optic disc was recorded. Values were adjusted for age.

A subset of patients (n = 32) had a clinical history of previous optic neuritis (ON) in at least one eye, 3 months or more before examination (ON group). The remaining subjects had never been affected by ON (n = 38, nON). One randomly chosen eye from subjects of nON group was included in the study. The ON-affected eye was chosen for subjects of the ON group.

Visual acuity was measured by a Snellen 20-foot wall chart. All subjects included had visual acuity values of 1.0 (Snellen equivalent of 20/20; with or without correction) of both eyes. Low Contrast Visual Acuity (LCVA) testing was performed using retroilluminated low-contrast Sloan letter charts (1.25% contrast at 2 m). Testing was performed by trained technicians experienced in examination of patients for research studies.

### Magnetic resonance imaging (MRI) data acquisition

Thirty-seven out of 114 MS patients had an MRI scan at 3T (Siemens Magnetom Allegra). The maximum gradient strength is 40 mTm1, with a maximum slew rate of 400mTm-1ms-1. The MRI session included for every subject: (1) a dual-echo turbo spin echo (TSE) (TR:6190 ms; TE1: 12 ms; TE2: 109 ms; echo train length [ETL]: 5; matrix: 256×192; field of view [FOV]: 230×172.5 mm2; 48 contiguous 3 mm thick slices) for lesion identification and segmentation (scan time: approximately 4 min); (2) a fluid attenuated inversion recovery (FLAIR) scan (TR: 8170 ms; TE:96; ms; Tl :2100 ms; ETL: 13; same FOV, matrix and number of slices as TSE) to use as a reference for lesion identification (scan time: 5 min); (3) morphological 3D T1-weighted magnetization prepared rapid acquisition gradient echo (MPRAGE) (TE = 2.74 ms, TR = 2500 ms, inversion time = 900 ms; flip angle = 8°; matrix = 256×208×176; FOV = 256×208×176mm3).

### MRI lesion segmentation

T2-hyperintense lesions were identified by consensus by two observers on the short echo images (proton density-weighted) of the TSE, for every patient. Lesions were outlined on the same scan using a semi-automated local thresholding contouring software (Jim 4.0, Xinapse System, Leicester, UK, http://www.xinapse.com/). FLAIR and T2-weighted scans were always used as a reference to increase confidence in lesion identification.

Voxel-based morphometry (VBM). The T1-weighted volumes (MPrage) were processed using the voxel-based morphometry protocol in SPM8 (http://www.fil.ion.ucl.ac.uk/spm/), an iterative combination of segmentations and normalizations to produce a grey matter (GM) probability map in standard space (Montreal Neurological Institute, or MNI coordinates) [22]. In order to compensate for compression or expansion which might occur during warping of images to match the template, GM maps were “modulated” by multiplying the intensity of each voxel in the final images by the Jacobian determinant derived from the deformation field [23]. GM maps were then smoothed using a 10-mm FWHM Gaussian kernel.

Statistical analysis was performed in SPM8, as detailed below.

For each tissue class (GM, white matter and CSF), every patient's global volume was estimated by integrating the intensity values over the whole segmented image. The following quantities were then derived: the total brain volume, computed as the sum of white and grey matter volume, the intracranial volume, computed as the sum of the total brain volume and the total CSF volume, and the brain parenchymal fraction (BPF), equal to the brain volume to intracranial volume ratio.

### Neuropsychological and disability assessment

All 114 patients underwent the neuropsychological and disability assessment by expert neurologists and neuropsychologists, who were blinded to laboratory and MRI results. All patients undergoing MRI examination were clinically and neuropsychologically examined within 1 week interval.

Expanded Disability Status Scale (EDSS) [24], a 10-point disease severity score derived from nine ratings for individual neurological domains, was administered to all MS patients by a trained and certified examining neurologist. Progression Index (PI) was defined as EDSS disease duration.

Cognitive functions were assessed using the Brief Repeatable Neuropsychological Battery (BRB) [25] and the Stroop Test (ST) [26]. The BRB assesses the cognitive domains most frequently impaired in MS subjects [27] and incorporates tests of verbal memory (SRT), visual memory (10/36 SPART), attention, concentration and speed of information processing (Paced Auditory Serial Addition Test [PASAT]; Symbol Digit Modality Test [SDMT]) and verbal fluency (WLG). Moreover, the ST was administered to evaluate frontal lobe executive functions, which are not assessed by the BRB [26]. Performance on each test of the BRB and on the ST was assessed by applying the available Italian normative values [28]. In particular, failure of a test was defined when the score was at least two standard deviations (SDs) below the mean normative values. Consistently with previous works [28], [29], those patients who failed at least three tests were considered CI (cognitive impaired), and those who failed less than three tests were considered CP (cognitive preserved). A grading system was applied to each patient's score on each cognitive test, dependent on the number of SDs below the normative mean (0: patient scored at or above normative mean; 1: patient scored ≤1 SD below normative mean; 2: patient scored >1 SD, but ≤2 SD below normative mean, etc.). The sum of these grades was determined across all variables to give the cognitive impairment index (CII), a single overall measure of cognitive impairment for each patient [30]–[32]. Cognitive tests were repeated after 18 months, using versions B of the BRB. Subjects with cognitive decline were defined those who had a CII change of ≥2 points from baseline.

Higher-order cognitive executive functions were assessed by Delis-Kaplan Executive Function System (D-KEFS) Sorting Test [33] in a subgroup of subjects (n = 62). It measures the examinee's ability of problem-solving behavior. In the free sorting condition, the examinee is presented with mixed-up cards that display both stimulus words and various perceptual features. The examinee is asked to sort the cards into two groups, according to as many different categorization rules, or concepts as possible and to describe the concepts used to generate each sort. In the sort recognition condition, the same sets of cards are each sorted by the examiner into two groups. We analyzed the total confirmed correct sorts, which represent the number of correct sorts for which the verbal description is awarded one or more points and the combined free description score (FDS), which is based on the sum of correct description scores in the free sorting and sort recognition condition [33], [34]. Scaled D-KEFS scores were used in the analysis. Depression was assessed through the Montgomery and Asberg Depression Rating Scale [35]. Subjects scoring moderate to severe depression were not included. No subject was taking psychoactive drugs or substances that might interfere with neuropsychological performance. Subjects were tested after at least 3 months from previous relapse and/or detection of active scans at MRI.

### Data analysis

According to our previous report [36] MS subjects were divided into two groups according to the AAT repeat polymorphism of CNR1 gene (short AAT: homozygous or heterozygous for allele with ≤11 repeats of AAT triplets; long AAT: homozygous for allele with ≥12 repeats of AAT triplets). Differences between two groups were analyzed using Student's t-test, Mann–Whitney test, and Fisher exact test, as appropriate. Correlation analysis was performed by calculating Spearman coefficients. Immunochemical data were reported as the mean ± standard error (SE) of independent determinations, each performed in duplicate. Other data were presented as mean ± SD. A p-value (p) of less than 0.05 was considered statistically significant. The average lesion load, brain volume and BPF were compared between groups using Student's t-tests. VBM statistical analysis was performed using a full factorial design, where a 2 level factor was used to model the group defined by the genotype and the lesion load. The interaction between group and lesion load was also modeled. Age and gender were entered as nuisance covariates to adjust for potential confounds. P-values were accepted as significant if lower than 0.05 familywise error rate (FWER), corrected for multiple comparisons at cluster level. Global GM volumes, derived from VBM segmentation, were also used to explore potential interactions between AAT repeat polymorphism of CNR1 gene and patients performance at the administered cognitive tests. Multivariate prognostic models were constructed for the cognitive performance as outcome. The association between genotype and both overall CI and specific impairment in different cognitive tests was assessed using multivariate binary logistic regression models. The association between genotype and cognitive performance as measured by both the overall CII and D-KEFS variables was assessed using multivariate linear regression models. Clinical and demographic variables (disease duration, age, gender, educational level, EDSS) and genotype were included as predictor variables in the two models. Age was not entered as potential confound in analysis of D-KEFS variables, because the values were already corrected for age. Finally, the assessment of the association between genotype and CI was replicated in the subgroup of subjects with shorter disease duration (years of disease <10) and less disability (EDSS <2.5). The association between genotype and cognitive decline was also assessed by performing a logistic regression model with disease duration, age, gender, educational level, EDSS, baseline CII and genotype as predictor variables. Two-way ANOVAs were performed to analyzed the main effects of two conditions (genotype versus CI or genotype versus ON) on the dependent variables (MRI or ophthalmologic variables) and their interactions. MRI was limited to 37 patients of the whole studied population, and safety concerns and claustrophobia were the main reasons to refuse MRI. Similarly, only 70 (21 also had MRI) out of 114 subjects accepted the ophthalmological assessment with OCT.

## Results

### CNR1 (AAT)n controls the expression level of CB_1_R in MS

**Figure 1:**
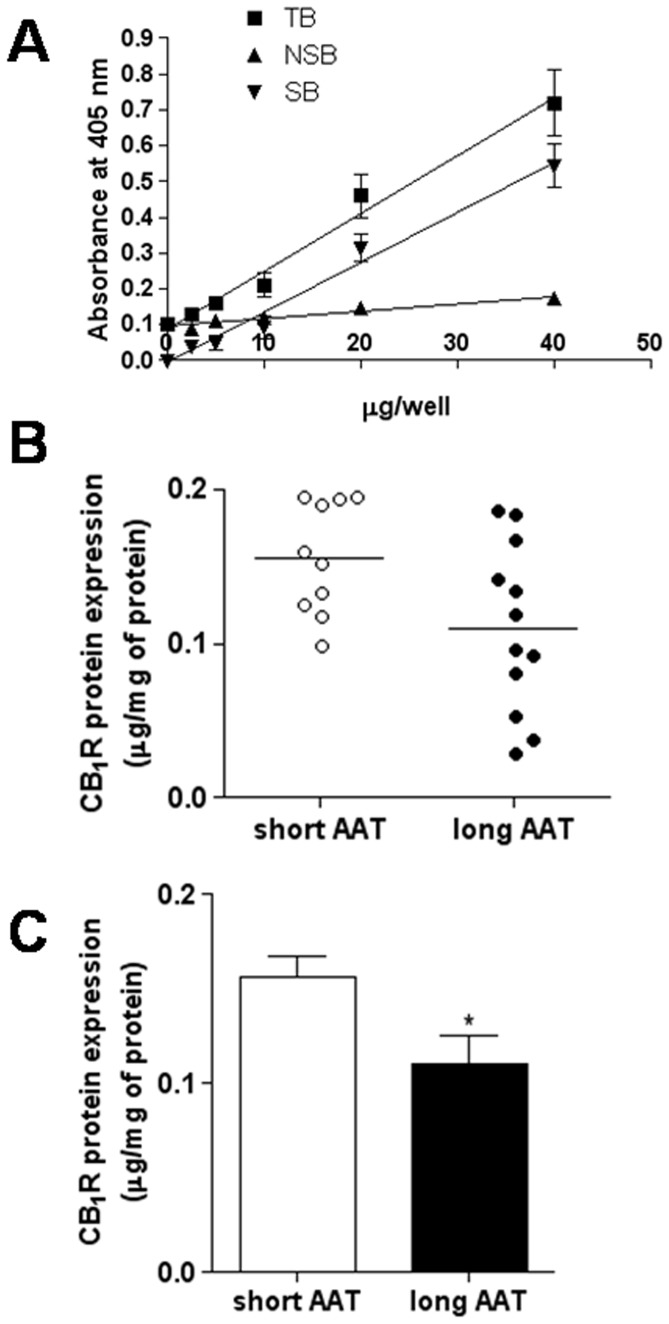
CB_1_R protein expression in MS lymphocytes. A. Calibration curve of the ELISA test with different concentrations of CHO-CB_1_ cell extracts. Specific binding (SB) of anti-CB_1_ antibody to CHO-CB_1_ cell extracts followed the equation y = 0.01133×+0.01951 (R^2^ = 0.9775). B. Dispersion graphs of CB_1_ protein content in human lymphocytes from short AAT group (white circles and bar) and long AAT group (black circles and bar) of patients. C. Histogram of cumulative data shown in panel B. *p = 0.0335.

To measure the impact of AAT repeats in the *CNR1* gene on CB_1_R expression, we generated in preliminary experiments a calibration curve with increasing amounts of CHO-CB_1_R cell extracts incubated with anti-CB_1_R antibodies. Total binding (TB) of cell extracts was quantified, and was compared to the nonspecific binding (NSB) of the same samples reacted with anti-CB_1_R antibodies that had been pre-incubated with the blocking peptide. NSB values were then subtracted from TB values, in order to calculate the specific binding (SB) of the cell extracts [21]. The dose-dependence curves of TB, NSB, and SB of different amounts of CHO-CB_1_R cell extracts are reported in Fig. 1A, that clearly shows the specificity of the anti-CB_1_R antibodies used in this study.

On this background, ELISA tests were used under the same conditions to calculate SB values, and hence CB_1_R content, in lymphocytes from the short (n = 10) and the long AAT group (n = 12). A significant (p = 0.0335) reduction of CB_1_R expression was observed in the long AAT group (CB_1_R content = 0.110±0.016 µg per mg of protein) with respect to the short AAT group (CB_1_R content = 0.156±0.011 µg per mg of total protein) (Fig. 1B,C).

### Clinical and genetic characteristics of MS patients

Nine allelic variants of the AAT repeats were found in the studied population. According to used nomenclature [36], allele 2 corresponded to (AAT)_8_, allele 3 to (AAT)_9_, allele 4 to (AAT)_10_, allele 5 to (AAT)_11_, allele 6 to (AAT)_12_, allele 7 to (AAT)_13_, allele 8 to (AAT)_14_, allele 9 to (AAT)_15_, allele 10 to (AAT)_16_. We failed to detect allele 1 in our sample. The alleles most frequently found were number 5 (26.7%), number 8 (25.0%) and number 9 (21.1%), in line with previous observations [36]. We performed the subsequent analysis classifying CNR1 alleles into a short and a long group, as previously reported [36].

The two genotype groups (short and long AAT) did not differ in terms of the main clinical and demographic characteristics (Table 1). Of note, PI (EDSS/disease duration) was higher in the long AAT group (0.53±0.45 versus 0.31±0.27; p<0.01), according to the previous reported association between (AAT)_n_ repeat polymorphism of CNR1 gene and disease progression in relapsing-remitting MS patients [36]. EDSS and disease duration were therefore taken into account as confounding factors during subsequent analyses.

**Table 1:** Demographic and clinical characteristics of MS subjects.

	total	short AAT	long AAT	p
Number	114	59	55	
gender (M/F)	38/76	19/40	19/36	ns
age (years)	36.8±9.2	37.1±9.3	36.6±9.1	ns
disease duration (years)	7.2±6.3	7.6±6.2	6.7±6.5	ns
EDSS	2.17±1.6	2.10±1.7	2.3±1.4	ns

ns, not significant; M, male; F, female; EDSS, Expanded Disability Status Scale.

### CNR1 (AAT)n influences the relationship between inflammation and neuronal damage in the optic nerve

**Figure 2:**
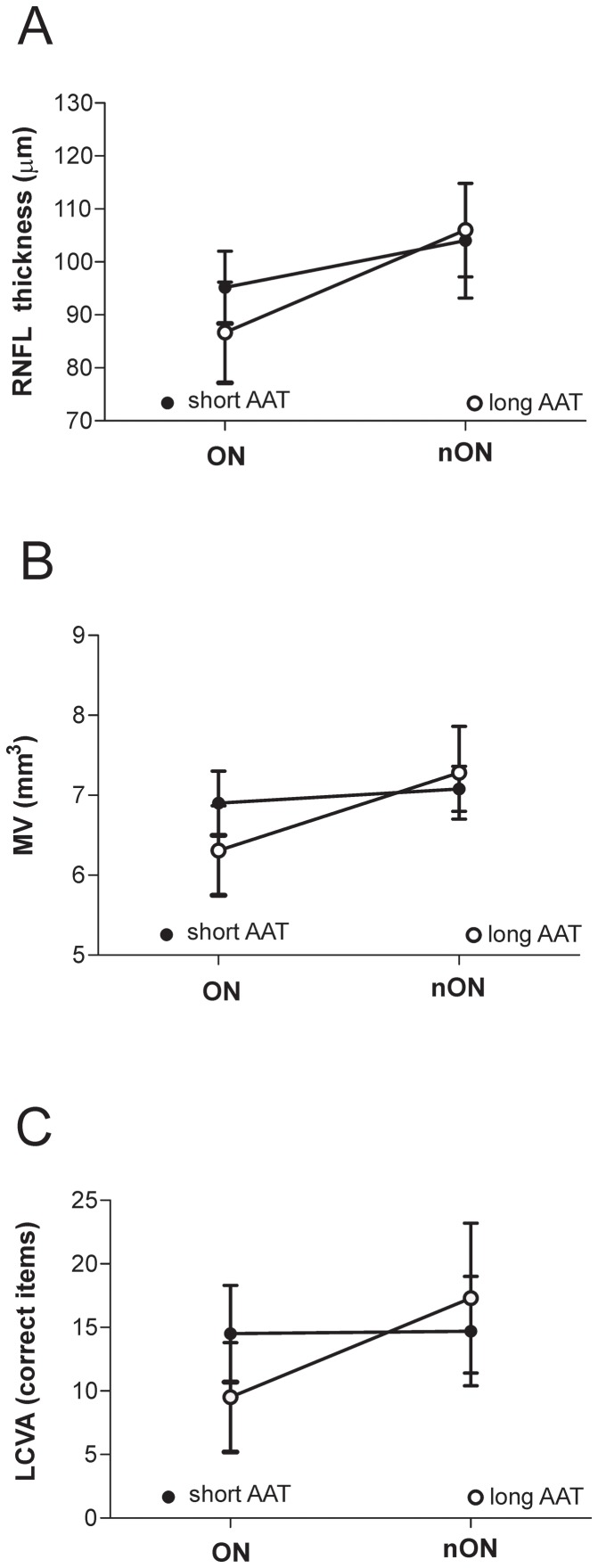
CNR1 (AAT)n influences the relationship between inflammation and neuronal atrophy in MS. A–C. Plot of interaction analysis between CNR1 genotype and previous optic neuritis (ON), analyzing RNFL thickness, MV and LCVA. A significant interaction between genotype and ON condition was found analyzing both RNFL thickness (A) and MV (B), confirming less severe neurodegenerative damage after inflammatory events in subjects with short AAT repeats. A significant interaction between the two conditions (genotype X ON) were also revealed by LCVA analysis (C), supporting the idea that the short AAT repeat genotype is associated with less severe visual impairment secondary to neuroinflammation in MS patients.

Axonal and neuronal loss in MS has been convincingly associated with reduced RNFL thickness and MV at the OCT. The availability of these noninvasive measures provides a unique potential for in vivo investigation of factors associated with axonal loss secondary to inflammatory demyelination or underlying primary neurodegeneration, by analyzing optic nerves from ON eyes or from unaffected eyes, respectively [37]–[41]. Thus, we investigated the possible relationship between genotype and OCT parameters in MS patients of both short and long AAT repeats groups. To evaluate the involvement of CNR1 polymorphism on the structural effects of an inflammatory insult to the optic nerve, MS patients were classified in those who had previously suffered from ON in at least one eye (n = 32) and those who had not (nON, n = 38), on the basis of their clinical history. Two-way ANOVAs were performed to analyzed the main effects of the two conditions (genotype versus ON) on the dependent variables (ophthalmologic variables) and their interactions. A significant main effect of ON condition was revealed analyzing both RNFL thickness (F = 39.86, p<0.0001) and MV (F = 25.26, p<0.0001), indicating a damage of neuronal structures after ON. Interestingly, subjects with short AAT repeats presented higher values of RNFL thickness and MV despite ON, suggesting less severe neurodegenerative damage after inflammatory events. In line with this, a significant interaction between genotype and ON condition was found (RNFL thickness: F = 5.57, p = 0.02; MV: F = 11.92, p = 0.001). Conversely, genotype per se failed to significantly affect OCT parameters (RNFL thickness: F = 2.13, p = 0.15; MV: F = 2.90, p = 0.09), confirming the selective involvement of CNR1 polymorphism in limiting axonal loss secondary to inflammatory demyelination but not primary neurodegeneration (Fig. 2A,B).

### CNR1 (AAT)n influences optic nerve function

Consistent results were obtained at the LCVA test, an emerging visual functional outcome [42]. In fact, a significant main effect (F = 13.71, p<0.001) of ON condition and a significant interaction (F = 12.37, p = 0.001) between the two conditions (genotype X ON) were revealed. In line with previous results, genotype failed to significantly affect LCVA (F = 1.12, p = 0.29) (Fig. 2C). These findings further support the idea that the short AAT repeat genotype is associated with less severe neuronal damage and visual impairment secondary to neuroinflammation in MS patients.

### CNR1 (AAT)n influences the relationship between lesion load and grey matter atrophy in MS brains

**Figure 3:**
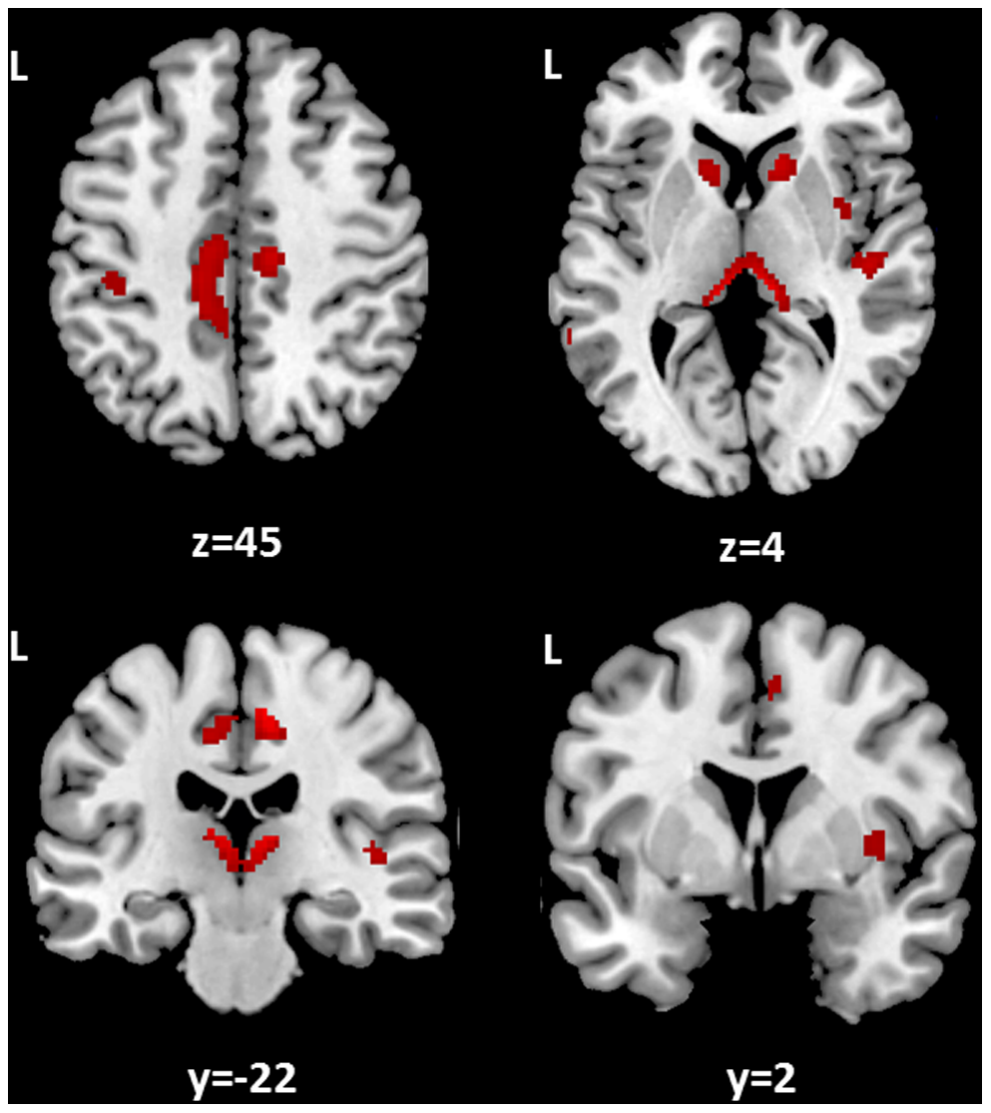
Association between lesion load and GM atrophy in MS brain. There are shown here in red, those brain areas in which local GM volumes are negatively associated with T2-weigthed lesion loads in the whole MS population. This association indicates that MS patients with larger lesion load tend to develop more GM atrophy in the thalamus, the head of the caudate nucleus and the cingulate cortex bilaterally, in the right insular cortex, and in the left postcentral gyrus. Statistical threshold: p values FWE-corrected <0.05. Spatial coordinates (x,y,z) in the figure are in MNI space.

**Figure 4:**
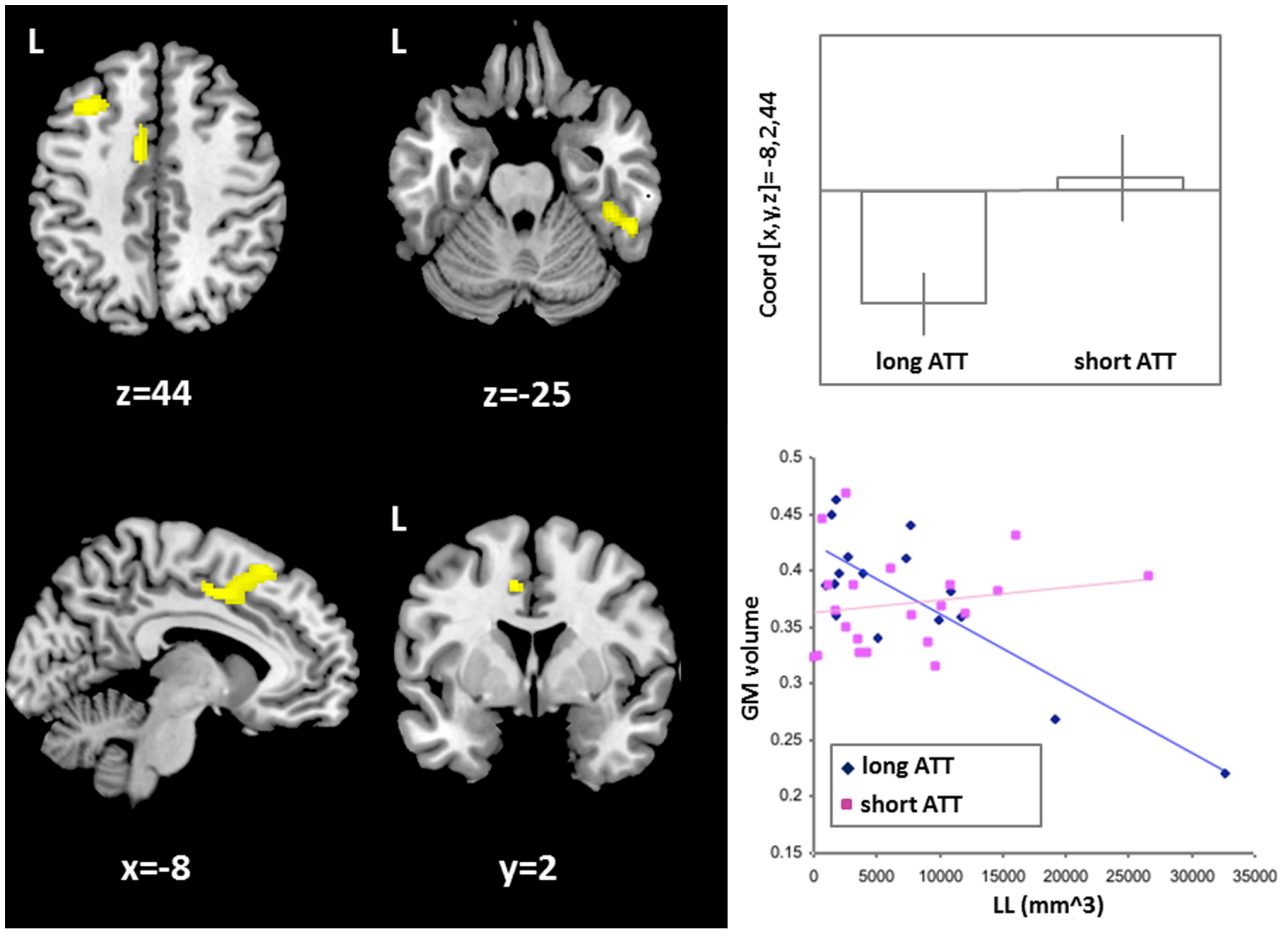
Interaction between the *CNR1* genotype and T2-weighted lesion loads on regional GM volumes. In yellow, there are shown those areas (i.e., left frontal and cingulate cortex, and right temporal cortex) in which an inverse correlation exists between T2-lesion load and local grey matter volumes in individuals with long AAT repeats, but that is absent in those with short AAT repeats. The plot on the top right shows this effect of interaction in the left frontal and cingulate area. This same effect is also confirmed, on a subject by subject basis, by the sctatterplot on the bottom right. Statistical threshold: p values FWE-corrected <0.05. Spatial coordinates (x,y,z) in the figure are in MNI space. Abbreviations: LL = T2-weighted lesion load; GM = grey matter.

In the population of patients who underwent MRI, no significant group differences were found between individuals who carried the short or the long ATT repeat with respect to mean age, lesion load, total brain volume, BPF, disease duration and gender distribution (Table 2). Also, no significant differences were found between the two patient groups in regional GM volumes (p<0.001, uncorrected). Conversely, a significant negative association (p<0.05, FWE-corrected) was found between regional GM volumes and lesion load (Fig. 3) across the whole population of subjects, indicating that MS patients with larger lesion load tend to develop more GM atrophy within the thalamus, the head of the caudate nucleus and the cingulate cortex bilaterally, in the right insular cortex, and in the left post-central gyrus. Interestingly, areas of significant (p<0.05, FWE corrected) group by lesion load interaction were found in the left frontal and cingulate cortex and in the right temporal cortex (Fig. 4, left). In those areas, the inverse correlation between lesion load and GM matter volume found in patients with the long AAT genotype was lost in those with short AAT repeats. These results indicate that, while in the former group patients with higher lesion load tend to develop more GM atrophy, in the second group this relationship breaks down, as expected for a relative preservation of selected GM areas in these subjects. This is also confirmed by the plot (top, right) and the scatterplot (bottom, right of Fig. 4).

**Table 2:** Demographic and clinical characteristics of subjects performing MRI study.

Subjects	All	Short AAT	Long AAT
N	37	21	16
Age (years)	36±9.3	37.5±10.3	34.1±7.7
M/F	9/28	5/15	4/12
EDSS median (range)	2.0 (0–6.0)	1.5 (0–6.0)	2.0 (0–6.0)
LL (mm^3^)	7234.45±7317.97	7537.40±6592.18	7003.62±8390.79
Brain volume (mL)	1049.3±119.0	1060.2±139.7	1041.1±103.4
BPF	0.658±0.066	0.645±0.073	0.667±0.059
Disease duration (years)	8.97±6.70	8.81±5.00	9.09±7.90

EDSS, Expanded Disability Status Scale; BPF, brain parechymal fraction; M, male; F, female; LL, Lesion Load. No significant between group difference was found for any of these variables.

### CNR1 (AAT)n regulates cognitive abilities in MS patients

**Figure 5:**
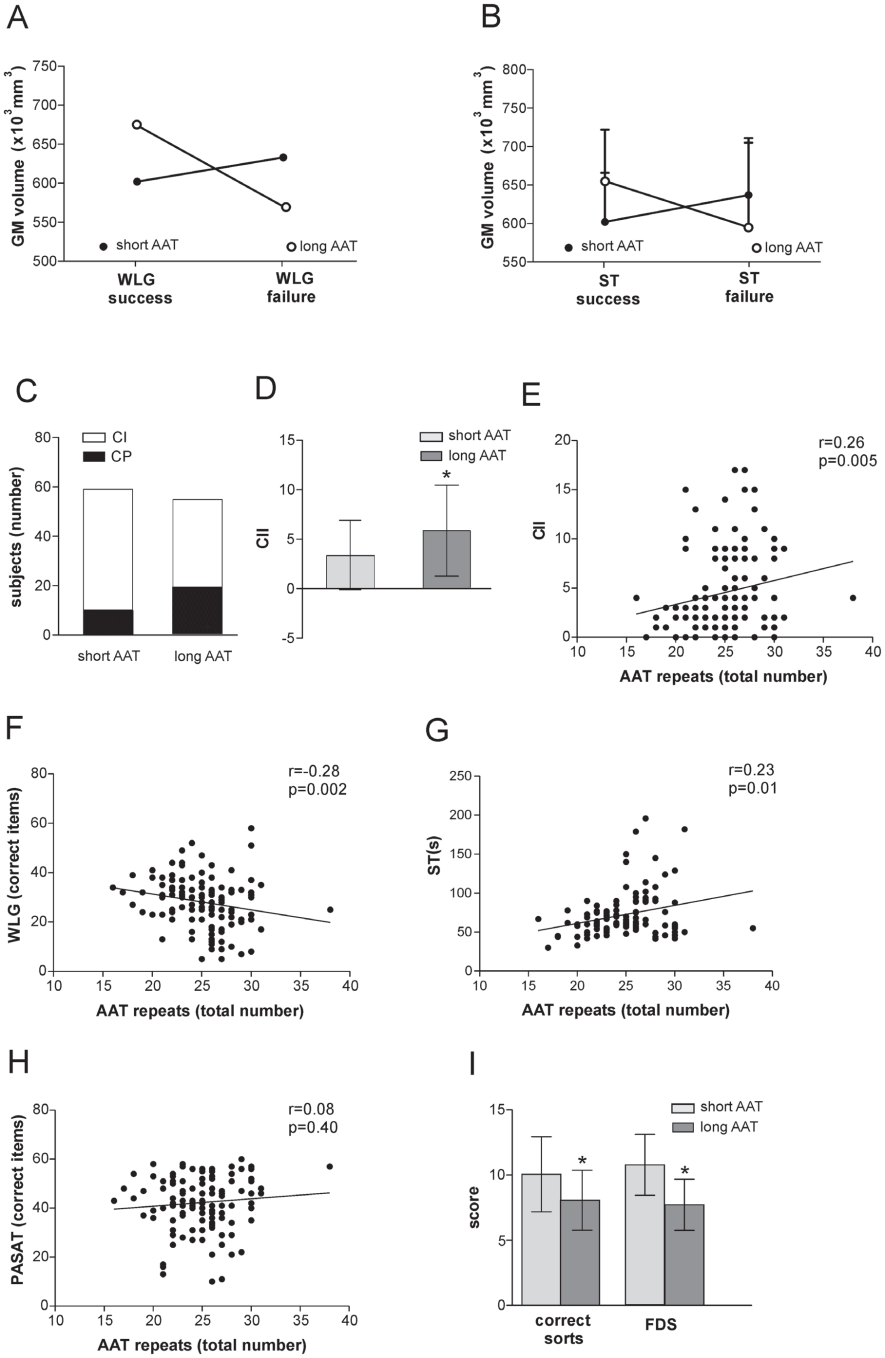
*CNR1* (AAT)n regulates cognitive abilities in MS patients. A, B. Plot of interaction analysis between CNR1 genotype and performance at WLG test (A) or ST (B), analyzing the GM volume. These data confirm the relative preservation of neuronal structures after inflammatory events in subjects with short AAT repeats. C. Cognitive impairment was more frequent in MS subjects homozygous for long (AAT)n repeats in CNR1 gene than short AAT repeats group. D. CII was higher among subjects of long AAT group. E–H. Correlation plots between CII (E), WLG (F), ST (G), PASAT (H) and the total number of AAT repeats on the two genes are shown to better demonstrate the association between the number of AAT repeats and cognitive performance. I. Subjects carrying short AAT repeats scored better at D-KEFS, an executive function test.

The global GM volume was lower, although not significantly, among subjects with cognitive impairment (CI: n = 15, 602.8±26.6 ml; CP: n = 22, 627.6±13.4 ml, p>0.05) and a non significant inverse correlation was found with CII (n = 37, r = −0.20, p>0.05). On the other hand, a detrimental role of the AAT long genotype on global GM volumes was found in subjects who failed specific tasks of executive functions. In fact a significant interaction between genotype and the failure of Word List Generation (WLG) test was revealed analyzing GM volumes, accounting for approximately 17.17% of the total variance (n = 37, F = 7.29, p = 0.01; Fig. 4A), suggesting the relative preservation of neuronal structures in subjects with short AAT repeats. In line with this, an interaction, despite non statically significant, was found between genotype and the failure of ST, accounting for approximately 8.35% of the total variance (n = 37, F = 3.03, p = 0.09; Fig. 5B). Conversely, no significant interactions were found on global GM volumes between genotype and failure of sustained attention tests (PASAT: n = 37, F:1.86, p = 0.18; SDMT: n = 37, F = 0.05, p = 0.82).

We further explored whether CNR1 (AAT)n polymorphism affects recruitment of cognitive-related networks by altering synaptic plasticity. MS patients were therefore classified as CI (n = 29)] or CP (n = 85) on the basis of their neuropsychological performance. CNR1 (AAT)n polymorphism was found to predict cognitive impairment, since the long AAT group had higher probability of failing more than two cognitive tests at equal values of age, disease duration, gender, education level and EDSS score. The response variable (overall CI in our model) was significantly affected also by disease duration (Table 3). Accordingly, CI subjects were more frequent in the long AAT group (Fig. 5C; p<0.05).

**Table 3:** Logistic regression (overall CI as response variable).

Variable	coefficient	SE	OR	95% confidence interval	p
genotype	1.31	0.52	3.70	1.32–10.34	0.01
EDSS	0.23	0.18	1.26	0.89–1.78	0.19
Age	−0.04	0.04	0.96	0.89–1.03	0.28
Gender	0.52	0.56	1.68	0.56–5.07	0.35
education	0.06	0.07	1.06	0.91–1.23	0.44
disease duration	0.17	0.06	1.18	1.05–1.33	0,004

SE, Standard Error; OR, Odds Ratio; EDSS, Expanded Disability Status Scale; CI, Cognitive Impairment.

The same results were obtained when overall cognitive performance was assessed through the CII independently of the presence of CI. CII was higher among subjects of the long AAT group (Fig. 5D) and related to genotype (F = 10.46, p = 0.001). Also disease duration (F = 14.59, p = 0.0002) and EDSS (F = 15.24, p = 0.0002) explained a considerable portion of the total variance.

CNR1 genotype and cognitive impairment were also associated to each other when the analysis was restricted to those patients with less remarkable disability (EDSS<2.5) and shorter disease duration (years of disease<10). Also in this subgroup of subjects (n = 62), in fact, genotype (coefficient: 2.70, SE: 1.08, odds ratio (OR): 14.87, p = 0.01) and disease duration (coefficient: 0.46, SE: 0.22, OR: 1.59, p = 0.03) predicted cognitive impairment. CII was confirmed to be related to genotype, explaining in this model the most portion of the total variance (genotype: F = 9.73, p = 0.003; disease duration: F = 4.77, p = 0.03; EDSS: F:2.81, p = 0.09).

Furthermore, CNR1 polymorphism was associated with higher risk of impairment on neuropsychological tests exploring executive functions (WLG and ST) but not on tasks of memory and sustained attention (Table 4). To better explore the association between the number of AAT repeats and cognitive performance, further analyses were performed using the total number of AAT repeats on the two genes for each subject. Of note, the overall CII, WLG and ST scores were found to be related to the total number of AAT repeats for each subject (Fig. 5E–G; n = 114; CII: r = 0.26, p = 0.005; WLG: r = −0.28, p = 0.002; ST: r = 0.23, p = 0.01). No significant correlations were found analyzing tests of sustained attention and memory (n = 114; PASAT: r = 0.08, p = 0.40, Fig. 5H; SDMT: r = 0.02, p = 0.86; Spatial Recall Test [SPART]: r = 0.05, p = 0.61; Selective Reminding Test [SRT]: r = −0.002, p = 0.97).

**Table 4:** Neuropsychological performance of MS subjects according to CNR1 (AAT)n polymorphism.

	proportion of failure (short/long AAT group)	OR	95% confidence interval	p
PASAT 3	13.5%/12.7%	1.02	0.29–3.59	0.96
PASAT 2	18.6%/16.4%	0.82	0.28–2.39	0.72
SDMT	23.7%/21.8%	1.00	0.39–2.80	0.99
SRT-LTS	8.5%/14.5%	1.27	0.41–3.90	0.67
SRT-CLTR	9.0%/12.7%	1.11	0.36–3.47	0.85
SRT-D	8.5%/12.7%	1.44	0.41–5.08	0.57
SPART	7.3%/8.2%	2.33	0.67–8.10	0.18
SPART-D	5.1%/14.5%	1.35	0.84–17.81	0.08
WLG	3.4%/20.0%	9.41	1.78–49.81	<0.001
ST	10.1%/45.4%	7.83	2.80–21.92	<0.001

PASAT, Paced Auditory Serial Addition Test; SDMT, Symbol Digit Modalities Test; SRT-LTS, Selective Reminding Test – Long Term Storage; SRT-CLTR, Selective Reminding Test – Consistent Long Term Retrieval; SRT-D, Selective Reminding Test – Delayed; SPART, Spatial Recall Test;SPART-D, Spatial Recall Test – Delayed; WLG, Word List Generation; ST, Stroop Test; OR, Odds Ratio.

To confirm the impact of CNR1 (AAT)n polymorphism on executive functions we also administered D-KEFS Sorting test to the subgroup of patients with shorter disease duration and less disability (n = 62). D-KEFS Sorting test is a standardized executive function test designed to assess higher cognitive functions [33]. Subjects with long AAT repeats had lower FDS and total confirmed correct sorts, both being related to genotype (FDS: F = 29.58, p<0.0001; correct sorts: F = 8.88, p = 0.004) (Fig. 5I).

Finally, the long AAT repeat genotype was found to predict cognitive decline, since this group had higher probability of worsening in CII at equal values of age, disease duration, gender, education level, EDSS score and baseline CII. The response variable was significantly affected also by age and baseline CII (Table 5).

**Table 5:** Logistic regression (CII worsening as response variable).

Variable	coefficient	SE	OR	95% confidence interval	p
genotype	1.67	0.68	5.30	1.40–19.98	0.01
disease duration	−0.04	0.06	0.95	0.85–1.07	0.45
EDSS	0.12	0.20	1.12	0.74–1.69	0.57
Age	0.14	0.04	1.15	1.06–1.26	0.001
Gender	0.15	0.62	1.15	0.34–3.92	0.81
education	0.14	0.09	1.15	0.96–1.38	0.13
baseline CII	0.19	0.08	1.22	1.04–1.42	0.01

CII, Cognitive Impairment Index; EDSS, Expanded Disability Status Scale; SE, Standard Error; OR, Odds Ratio.

## Discussion

Candidate gene association studies are potentially useful in determining genetic influences on disease progression in MS and a neuropsychological outcome measure may more closely relate to the burden of disease than measures of physical disease progression. We have already reported that the long alleles of (AAT)_n_ repeat polymorphism of CNR1 gene (>11 repeats) represent a genetic risk factor for disease progression in relapsing-remitting MS [36].

Here, we have provided initial information on the biological impact of this polymorphism on CB_1_R protein expression in a sample of MS patients, and we have addressed its functional consequences on inflammation-induced optic nerve and brain structural damage, as well as on visual and cognitive functioning. To strengthen our conclusions, however, the impact of CNR1 (AAT)_n_ repeat polymorphism on CB_1_R mRNA levels should also be addressed, along with measurements in a larger independent population of MS patients, by using other CB_1_R-specific antibodies, and in healthy individuals. Based on the results of the present investigation, in fact, we cannot exclude that the (AAT)_n_ repeat polymorphism affects CB_1_R function only in the context of MS disease, and not in control subjects. Brain inflammation, in fact, can reduce per se CB_1_R function in EAE mice [43], raising the possibility that long (AAT)_n_ repeats within the CNR1 gene only impact on the effects of the inflammatory milieu, likely interleukin-1ß [44], on CB_1_R expression and function. Also this concept, however, requires further experimental work.

The hypothesis that CNR1 (AAT)n influences the relationship between inflammation and neuronal atrophy in MS was first demonstrated by OCT evaluation. OCT provides noninvasive means to quantify the structural effects of an inflammatory insult to the optic nerve, which can then be compared to functional outcomes, to construct a structural-functional paradigm of central nervous system (CNS) injury [39]. In fact, OCT can be used to measure the RNFL thickness and the MV, that are both reduced following the development of MS and ON [38], [45,present work], and can therefore be used as a correlate of global axonal loss [45].

Changes in RNFL thickness after ON have been interpreted to reflect initial axoplasmic flow stasis and subsequent attrition caused by inflammation in the anterior visual pathway. Recent studies have shown that the extent of RNFL atrophy correlates with MRI measures of optic nerve, brain atrophy and disease activity in MS patients [41], [46], [47]. Here we showed a significant association between CNR1 genotype and ON condition, analyzing the extent of RNFL thickness and MV reduction, thus indicating the relative preservation of neuronal structures after inflammatory events in subjects with short AAT repeats. Finally, similar results were obtained analyzing the LCVA test, an emerging visual functional outcome [42]. These findings are consistent with the idea that short AAT repeats in CNR1 gene favor the functional compensation to the neuronal loss secondary to inflammation in the CNS of MS patients.

Brain tissue damage presents in MS with macroscopic white matter lesions, as well as with microscopic damage to the so-called normal appearing white (NAWM) and grey matter (NAGM) [48], [49]. Damage to the GM is known to be due to either the accumulation of cortical MS lesions or to the occurrence of Wallerian degeneration secondary to white matter (WM) damage. GM damage has been found to play a critical role in determining motor and cognitive disabilities in MS patients, and in accounting (at least partially) for the progression from relapsing to secondary progressive MS course. In the current study, using VBM for regional GM volumetrics, we found an expected direct association between lesion load and accumulation of regional GM atrophy, when considering our MS patients as a whole group. Within the limitations of the cross-sectional design of this study, and taking into account the relatively small sample, this confirms that in MS a relevant proportion of GM damage is explained by accumulation of macroscopic WM lesions, and the consequent Wallerian degeneration of GM. However, when considering the interaction between the genetic pattern of AAT repeats and the relationship between lesion load and regional GM volumes, we found that the presence of short AAT repeats had a significant impact on breaking down this relationship in several cortical locations. This suggests that the presence of short AAT repeats reduces the WM damage in MS brains, and its contribution in determining secondary degeneration of the GM tissue.

Interestingly, the set of GM regions, whose atrophy was found to be attenuated by the presence of short AAT repeats, includes areas with a major role in cognition, and in fact we also found that MS patients with short AAT repeats had less cognitive impairment than the long AAT patients. Accordingly, we have found that long AAT MS subjects had a major risk to develop cognitive impairment and to incur into cognitive decline, at equal values of age, disease duration, gender, education level and physical disability. The same results were obtained when overall cognitive performance was assessed through the CII independently of the presence of cognitive impairment. Therefore, we confirmed our results studying the relationship between CNR1 alleles and cognitive performance in a condition in which possible confounders, such as disease duration and disability levels, may have a minor impact.

MS-related cognitive impairment has been consistently associated with brain atrophy also in the earliest disease stages [27], [50], [51], and damage to several GM structures can be associated with impairment of specific cognitive functions [52]. Here we have demonstrated that variants of *CNR1* gene have a direct effect on executive functioning measured by WLG test, ST (inhibition of automatic response), D-KEFS Sorting test (verbal/nonverbal modality-specific problem-solving skills, ability to transfer sorting concepts into action and ability to inhibit previous description responses to engage in flexibility of thinking). Damage to the frontal and prefrontal cortex leads to impairment in executive functioning, which normally allows individuals to effectively engage in complex goal-directed behaviors [53]–[55]. Of note, the contribution of inflammatory WM damage in determining secondary degeneration of the GM tissue was attenuated in subjects with short AAT repeats at the level of cerebral areas involved in these processes. In line with this, these subjects scored better in executive function tests than the long AAT group. Of note, the extent of GM atrophy was not significantly related to global cognitive impairment in our sample, but a clear interaction between genotype and cognitive performance was found by analyzing GM volume, suggesting the relative preservation of neuronal structures in subjects with short AAT repeats.

To produce cognitive performance similar to healthy controls, MS patients require greater recruitment of prefrontal cortical regions [56], [57] and greater deactivation of the anterior cingulate cortex [57], the core components of the brain's default network, which consists of brain regions more active during rest or passive thought than directed cognitive processing. Among healthy individuals, prefrontal recruitment is positively associated with age [58]. Therefore, the major extent of GM atrophy secondary to neuroinflammation observed among subjects homozygous for long AATn (present work) might disrupt the activation of cerebral networks essential to limit the negative impact of brain disease on cognition.

In conclusion, our study points to CB_1_R as an interesting molecular target for preventing neuronal loss and cognitive impairment in MS as well as in other CNS disorders in which inflammation-driven neurodegeneration process play a role.

## References

[1] Howlett AC, Blume LC, Dalton GD (2010) CB1 cannabinoid receptors and their associated proteins. Curr Med Chem 17: 1382–1393. 20166926 10.2174/092986710790980023 PMC3179980

[2] Katona I (2009) Endocannabinoid receptors: CNS localization of the CB1 cannabinoid receptor. Curr Top Behav Neurosci 1: 65–86. 21104380 10.1007/978-3-540-88955-7_3

[3] Pertwee RG, Howlett AC, Abood ME, Alexander SP, Di Marzo V, et al (2010) International Union of Basic and Clinical Pharmacology LXXIX Cannabinoid receptors and their ligands: beyond CB1 and CB2. Pharmacol Rev 62: 588–631. 21079038 10.1124/pr.110.003004 PMC2993256

[4] Katona I, Freund TF (2008) Endocannabinoid signaling as a synaptic circuit breaker in neurological disease. Nat Med 14: 923–930. 18776886 10.1038/nm.f.1869

[5] Arévalo-Martín A, Molina-Holgado E, Guaza C (2012) A CB1/CB2 receptor agonist, WIN 55,212-2, exerts its therapeutic effect in a viral autoimmune model of multiple sclerosis by restoring self-tolerance to myelin. Neuropharmacology 63: 385–393. 22561283 10.1016/j.neuropharm.2012.04.012

[6] Reguero L, Puente N, Elezgarai I, Mendizabal-Zubiaga J, Canduela MJ, et al (2011) GABAergic and cortical and subcortical glutamatergic axon terminals contain CB1 cannabinoid receptors in the ventromedial nucleus of the hypothalamus. PLoS One 6: e26167. 22022550 10.1371/journal.pone.0026167 PMC3191179

[7] Bisogno T, Di Marzo V (2010) Cannabinoid receptors and endocannabinoids: role in neuroinflammatory and neurodegenerative disorders. CNS Neurol Disord Drug Targets 9: 564–753. 20632970 10.2174/187152710793361568

[8] Saito VM, Rezende RM, Teixeira AL (2012) Cannabinoid modulation of neuroinflammatory disorders. Curr Neuropharmacol 10: 159–166. 23204985 10.2174/157015912800604515 PMC3386505

[9] Scotter EL, Abood ME, Glass M (2010) The endocannabinoid system as a target for the treatment of neurodegenerative disease. Br J Pharmacol 160: 480–498. 20590559 10.1111/j.1476-5381.2010.00735.x PMC2931550

[10] Lou ZY, Zhao CB, Xiao BG (2012) Immunoregulation of experimental autoimmune encephalomyelitis by the selective CB1 receptor antagonist. J Neurosci Res 90: 84–95. 21922514 10.1002/jnr.22721

[11] Pryce G, Ahmed Z, Hankey DJ, Jackson SJ, Croxford JL, et al (2003) Cannabinoids inhibit neurodegeneration in models of multiple sclerosis. Brain 126: 2191–2202. 12876144 10.1093/brain/awg224

[12] Rossi S, Furlan R, De Chiara V, Muzio L, Musella A, et al (2011) Cannabinoid CB1 receptors regulate neuronal TNF-α effects in experimental autoimmune encephalomyelitis. Brain Behav Immun 25: 1242–1248. 21473912 10.1016/j.bbi.2011.03.017

[13] Forder JP, Tymianski M (2009) Postsynaptic mechanisms of excitotoxicity: involvement of postsynaptic density proteins, radicals, and oxidant molecules. Neuroscience 158: 293–300. 19041375 10.1016/j.neuroscience.2008.10.021

[14] Mandolesi G, Grasselli G, Musumeci G, Centonze D (2010) Cognitive deficits in experimental autoimmune encephalomyelitis: neuroinflammation and synaptic degeneration. Neurol Sci 31: S255–S259. 20635112 10.1007/s10072-010-0369-3

[15] Rossi S, Furlan R, De Chiara V, Motta C, Studer V, et al (2012) Interleukin-1ß causes synaptic hyperexcitability in multiple sclerosis. Ann Neurol 71: 76–83. 22275254 10.1002/ana.22512

[16] Zhang PW, Ishiguro H, Ohtsuki T, Hess J, Carillo F, et al (2004) Human cannabinoid receptor 1: 5′ exons, candidate regulatory regions, polymorphisms, haplotypes and association with polysubstance abuse. Mol Psychiatry 9: 916–931. 15289816 10.1038/sj.mp.4001560

[17] Li YC, Korol AB, Fahima T, Nevo E (2004) Microsatellites within genes: structure, function, and evolution. Mol Biol Evol 21: 991–1007. 14963101 10.1093/molbev/msh073

[18] Ramil E, Sánchez AJ, González-Pérez P, Rodríguez-Antigüedad A, Gómez-Lozano N, et al (2010) The cannabinoid receptor 1 gene (CNR1) and multiple sclerosis: an association study in two case-control groups from Spain. Mult Scler 16: 139–146. 20007426 10.1177/1352458509355071

[19] Polman CH, Reingold SC, Edan G, Filippi M, Hartung HP, et al (2005) Diagnostic criteria for multiple sclerosis: 2005 revisions to the “McDonald Criteria”. Ann Neurol 58: 840–846. 16283615 10.1002/ana.20703

[20] Cencioni MT, Chiurchiù V, Catanzaro G, Borsellino G, Bernardi G, et al (2010) Anandamide suppresses proliferation and cytokine release from primary human T-lymphocytes mainly via CB2 receptors. PLoS One 5: e8688. 20098669 10.1371/journal.pone.0008688 PMC2809084

[21] Catani MV, Gasperi V, Catanzaro G, Baldassarri S, Bertoni A, et al (2010) Human platelets express authentic CB1 and CB2 receptors. Curr Neurovasc Res 7: 311–318. 20854251 10.2174/156720210793180774

[22] Ashburner J, Friston KJ (2005) Unified segmentation. Neuroimage 26: 839–851. 15955494 10.1016/j.neuroimage.2005.02.018

[23] Ashburner J, Friston KJ (2001) Why voxel-based morphometry should be used. Neuroimage 14: 1238–1243. 11707080 10.1006/nimg.2001.0961

[24] Kurtzke JF (1983) Rating neurologic impairment in multiple sclerosis: An Expanded Disability Status Scale EDSS. Neurology 33: 1444–1452. 6685237 10.1212/wnl.33.11.1444

[25] Rao SM, the Cognitive Function Study Group of the National Multiple Sclerosis Society (1990) A manual for the Brief Repeatable Battery of Neuropsychological Tests in Multiple Sclerosis. Milwaukee: Medical College of Wisconsin.

[26] Barbarotto R, Laiacona M, Frosio R, Vecchio M, Farinato A, et al (1998) A normative study on visual reaction times and two Stroop colour-word tests. Ital J Neurol Sci 19: 161–170. 10933471 10.1007/BF00831566

[27] Amato MP, Bartolozzi ML, Zipoli V, Portaccio E, Mortilla M, et al (2004) Neocortical volume decrease in relapsing–remitting MS patients with mild cognitive impairment. Neurology 63: 89–93. 15249616 10.1212/01.wnl.0000129544.79539.d5

[28] Amato MP, Portaccio E, Goretti B, Zipoli V, Ricchiuti L, et al (2006) The Rao's Brief Repeatable Battery and Stroop Test: Normative values with age, education and gender corrections in an Italian population. Mult Scler 12: 787–793. 17263008 10.1177/1352458506070933

[29] Amato MP, Zipoli V, Goretti B, Portaccio E, De Caro MF, et al (2006) Benign multiple sclerosis: cognitive, psychological and social aspects in a clinical cohort. J Neurol 253: 1054–1059. 16609810 10.1007/s00415-006-0161-8

[30] Camp SJ, Stevenson VL, Thompson AJ, Miller DH, Borras C, et al (1999) Cognitive function in primary progressive and transitional progressive multiple sclerosis: a controlled study with MRI correlates. Brain 122: 1341–1348. 10388799 10.1093/brain/122.7.1341

[31] Patti F (2009) Cognitive impairment in multiple sclerosis. Mult Scler 15: 2–8. 18805842 10.1177/1352458508096684

[32] Patti F, Amato MP, Trojano M, Bastianello S, Tola MR, et al (2009) Cognitive impairment and its relation with disease measures in mildly disabled patients with relapsing-remitting multiple sclerosis: baseline results from the Cognitive Impairment in Multiple Sclerosis COGIMUS study. Mult Scler 15: 779–788. 19542262 10.1177/1352458509105544

[33] Delis DC, Kramer JH, Kaplan E, Holdnack J (2004) Reliability and validity of the Delis-Kaplan Executive Function System: an update. J Int Neuropsychol Soc 10: 301–303. 15012851 10.1017/S1355617704102191

[34] Krueger F, Pardini M, Huey ED, Raymont V, Solomon J, et al (2011) The role of the Met66 brain-derived neurotrophic factor allele in the recovery of executive functioning after combat-related traumatic brain injury. J Neurosci 31: 598–606. 21228168 10.1523/JNEUROSCI.1399-10.2011 PMC3195417

[35] Montgomery SA, Asberg M (1979) A new depression scale designed to be sensitive to change. Br J Psychiatry 134: 382–389. 444788 10.1192/bjp.134.4.382

[36] Rossi S, Buttari F, Studer V, Motta C, Gravina P, et al (2011) The AATn repeat of the cannabinoid CB1 receptor gene influences disease progression in relapsing multiple sclerosis. Mult Scler 17: 281–288. 21148019 10.1177/1352458510388680

[37] Barkhof F, Calabresi PA, Miller DH, Reingold SC (2009) Imaging outcomes for neuroprotection and repair in multiple sclerosis trials. Nat Rev Neurol 5: 256–266. 19488083 10.1038/nrneurol.2009.41

[38] Burkholder BM, Osborne B, Loguidice MJ, Bisker E, Frohman TC, et al (2009) Macular volume determined by optical coherence tomography as a measure of neuronal loss in multiple sclerosis. Arch Neurol 66: 1366–1372. 19901168 10.1001/archneurol.2009.230

[39] Costello F (2011) Evaluating the use of optical coherence tomography in optic neuritis. Mult Scler Int 2011: 148394. 22096626 10.1155/2011/148394 PMC3196333

[40] Frohman EM, Fujimoto JG, Frohman TC, Calabresi PA, Cutter G, et al (2008) Optical coherence tomography: a window into the mechanisms of multiple sclerosis. Nat Clin Pract Neurol 4: 664–675. 19043423 10.1038/ncpneuro0950 PMC2743162

[41] Gordon-Lipkin E, Chodkowski B, Reich DS, Smith SA, Pulicken M, et al (2007) Retinal nerve fiber layer is associated with brain atrophy in multiple sclerosis. Neurology 69: 1603–1609. 17938370 10.1212/01.wnl.0000295995.46586.ae

[42] Baier ML, Cutter GR, Rudick RA, Miller D, Cohen JA, et al (2005) Low-contrast letter acuity testing captures visual dysfunction in patients with multiple sclerosis. Neurology 64: 992–995. 15781814 10.1212/01.WNL.0000154521.40686.63

[43] Centonze D, Bari M, Rossi S, Prosperetti C, Furlan R, et al (2007) The endocannabinoid system is dysregulated in multiple sclerosis and in experimental autoimmune encephalomyelitis. Brain 130: 2543–2553. 17626034 10.1093/brain/awm160

[44] Rossi S, Sacchetti L, Napolitano F, De Chiara V, Motta C, et al (2012) Interleukin-1ß causes anxiety by interacting with the endocannabinoid system. J Neurosci 32: 13896–13905. 23035099 10.1523/JNEUROSCI.1515-12.2012 PMC6704788

[45] Katie L, David B (2012) Optical Coherence Tomography Detection of Neurodegeneration in Multiple Sclerosis. CNS Neurol Disord Drug Targets 11: 518–527. 22583437 10.2174/187152712801661185

[46] Sepulcre J, Murie-Fernandez M, Salinas-Alaman A, García-Layana A, Bejarano B, et al (2007) Diagnostic accuracy of retinal abnormalities in predicting disease activity in MS. Neurology 68: 1488–1494. 17470751 10.1212/01.wnl.0000260612.51849.ed

[47] Trip SA, Schlottmann PG, Jones SJ, Li WY, Garway-Heath DF, et al (2006) Optic nerve atrophy and retinal nerve fibre layer thinning following optic neuritis: evidence that axonal loss is a substrate of MRI-detected atrophy. Neuroimage 31: 286–293. 16446103 10.1016/j.neuroimage.2005.11.051

[48] Akbar N, Lobaugh NJ, O'Connor P, Moradzadeh L, Scott CJ, et al (2010) Diffusion tensor imaging abnormalities in cognitively impaired multiple sclerosis patients. Can J Neurol Sci 37: 608–614. 21059506 10.1017/s0317167100010775

[49] Moll NM, Rietsch AM, Thomas S, Ransohoff AJ, Lee JC, et al (2011) Multiple sclerosis normal-appearing white matter: pathology-imaging correlations. Ann Neurol 70: 764–773. 22162059 10.1002/ana.22521 PMC3241216

[50] Amato MP, Portaccio E, Goretti B, Zipoli V, Battaglini M, et al (2007) Association of neocortical volume changes with cognitive deterioration in relapsing-remitting multiple sclerosis. Arch Neurol 64: 1157–1161. 17698706 10.1001/archneur.64.8.1157

[51] Lanz M, Hahn HK, Hildebrandt H (2007) Brain atrophy and cognitive impairment in multiple sclerosis: a review. J Neurol 254: II43–II48. 17503128 10.1007/s00415-007-2011-8

[52] Horakova D, Kalincik T, Dusankova JB, Dolezal O (2012) Clinical correlates of grey matter pathology in multiple sclerosis. BMC Neurol 12: 10. 22397707 10.1186/1471-2377-12-10 PMC3311149

[53] Bamdad MJ, Ryan LM, Warden DL (2003) Functional assessment of executive abilities following traumatic brain injury. Brain Inj 17: 1011–1020. 14555361 10.1080/0269905031000110553

[54] Fine EM, Delis DC, Dean D, Beckman V, Miller BL, et al (2009) Left frontal lobe contributions to concept formation: a quantitative MRI study of performance on the Delis-Kaplan Executive Function System Sorting Test. J Clin Exp Neuropsychol 31: 624–631. 19031322 10.1080/13803390802419017 PMC2743528

[55] Huey ED, Goveia EN, Paviol S, Pardini M, Krueger F, et al (2009) Executive dysfunction in frontotemporal dementia and corticobasal syndrome. Neurology 72: 453–459. 19188577 10.1212/01.wnl.0000341781.39164.26 PMC2677529

[56] Forn C, Barros-Loscertales A, Escudero J, Benlloch V, Campos S, et al (2007) Compensatory activations in patients with multiple sclerosis during preserved performance on the auditory N-back task. Hum Brain Mapp 28: 424–430. 16944483 10.1002/hbm.20284 PMC6871480

[57] Sweet LH, Rao SM, Primeau M, Durgerian S, Cohen RA (2006) Functional magnetic resonance imaging response to increased verbal working memory demands among patients with multiple sclerosis. Hum Brain Mapp 27: 28–36. 16001441 10.1002/hbm.20163 PMC6871410

[58] Rypma B, Berger JS, Genova H, Rebbechi D, D'Esposito M (2005) Dissociating age-related changes in cognitive strategy and neural efficiency using event-related fMRI. Cortex 41: 582–594. 16042034 10.1016/s0010-9452(08)70198-9

